# Functional Bacterial Cellulose-Based MXene (Ti_3_C_2_T*_x_*) Electronic-Skin Patch for Accelerated Healing and Monitoring

**DOI:** 10.34133/bmef.0109

**Published:** 2025-03-11

**Authors:** Saliha Nur lIhan, Bahar Akyuz Yilmaz, Fatih Ciftci

**Affiliations:** ^1^ Department of Biomedical Engineering, Fatih Sultan Mehmet Vakıf University, Istanbul, Turkey.; ^2^ Faculty of Engineering, Department of Computer Engineering, Fatih Sultan Mehmet Vakıf University, Istanbul, Turkey.; ^3^ Faculty of Science, Department of Molecular Biology and Genetics, Aksaray University, Aksaray, Turkey.; ^4^ Department of Technology Transfer Office, Fatih Sultan Mehmet Vakıf University, Istanbul, Turkey.

## Abstract

**Objective:** This study aims to develop and characterize electroactive hydrogels based on reduced bacterial cellulose (BC) and Ti_3_C_2_T*_x_*-MXene for their potential application in wound healing and real-time monitoring. **Impact Statement:** The integration of Ti_3_C_2_T*_x_*-MXene into BC matrices represents a novel approach to creating multifunctional hydrogels that combine biocompatibility, electrical conductivity, and mechanical durability. These properties make the hydrogels promising candidates for advanced wound care and real-time monitoring applications. **Introduction:** Wound healing requires materials that support cell growth, promote tissue regeneration, and enable real-time monitoring. MXenes, a class of 2-dimensional materials, offer unique electrical and mechanical properties, making them suitable for biomedical applications. This study explores the integration of Ti_3_C_2_T*_x_*-MXene with BC, a biopolymer known for its excellent biocompatibility and mechanical strength, to create electroactive composite hydrogel films for advanced wound care. **Methods:** Ti_3_C_2_T*_x_*-MXene was synthesized by etching Ti_3_AlC_2_ with hydrofluoric acid and integrated into BC pellicles produced by *Gluconacetobacter xylinum*. The composite hydrogel films underwent characterization through x-ray diffraction (XRD), x-ray photoelectron spectroscopy (XPS), Fourier transform infrared spectroscopy (FTIR), and thermogravimetric analysis (TGA) to determine structural, chemical, and thermal properties. Mechanical testing assessed tensile and compressive strengths. Biological assessments, including cell viability, hemolysis rate, and protein expression, evaluated biocompatibility and regenerative potential. **Results:** XRD confirmed the crystallographic structure of MXene and BC composite film. XPS and FTIR validated the successful incorporation of MXene into the film matrix. Composite hydrogel films demonstrated a tensile strength of 3.5 MPa and a compressive strength of 4.2 MPa. TGA showed stability up to 350 °C, and the electrical conductivity reached 9.14 × 10^−4^ S/m, enabling real-time monitoring capabilities. Cell viability exceeded 95%, with a hemolysis rate below 2%. Protein expression studies revealed the ability to promote skin regeneration through collagen I, K10, K5, and filaggrin expression. **Conclusion:** The BC/MXene composite hydrogel films exhibit important potential as electronic-skin patches for accelerating wound healing and enabling real-time monitoring. Their unique combination of mechanical durability, electrical conductivity, and biocompatibility highlights their promise for advanced wound care applications.

## Introduction

The development of biomimetic materials for tissue engineering and wound healing has become a crucial need in modern medicine [1–3]. These materials are expected to be biocompatible, mechanically robust, and biodegradable, supporting cell proliferation, tissue regeneration, and accelerated wound healing. In recent years, bacterial cellulose (BC) and MXene materials have demonstrated important potential for such applications. BC, produced by bacteria like *Gluconacetobacter xylinum*, is a natural polymer that exhibits exceptional mechanical strength, high water retention capacity, and excellent biocompatibility [4,5]. These attributes make BC an ideal candidate for both wound healing materials and biomimetic tissue scaffolds [6,7]. Furthermore, BC’s ability to support cell adhesion and proliferation has drawn considerable attention for its applications in tissue engineering. When BC-based wound dressings were developed, BC could not affect the behavior of electrically sensitive cells on its own because it could not conduct electricity [8]. For this reason, the creation of composites with new-generation products with high electrical conductivity has come to the fore in recent years [9,10].

MXenes, particularly Ti_3_C_2_T*_x_*-MXene, are recognized for their outstanding electrical conductivity, mechanical resilience, and flexibility [11–13]. The electrical conductivity of MXenes introduces the possibility of enhancing cellular processes, especially in electroactive tissue engineering applications, by accelerating the healing process. The use of MXene materials in biomedical applications addresses the need for electrical stimulation (ES) during wound healing while simultaneously enhancing the structural function of tissue scaffolds. BC/MXene composites have attracted great interest in recent years due to their promising applications in various fields such as energy storage, sensors, and environmental cleanup. Several studies have reported that these composites exhibit impressive properties in flexible electronics [11], supercapacitors [10,14–16], water purification systems [17–20], and tissue engineering [21]. These findings demonstrate the great potential of BC/MXene composites in the field of sustainable technology. The combination of these two materials BC and MXene-based composite hydrogel films presents a revolutionary solution especially for tissue engineering and wound healing [21–23]. This composite material integrates the natural, biomimetic properties of BC, which support tissue regeneration, with the electrical conductivity of MXene, which may accelerate cellular processes [24,25]. In wound care applications, these composite hydrogel films are designed to maintain moisture balance, support cell proliferation, and, through their electroactive properties, facilitate cellular healing.

This study aims to leverage the synergistic effects of BC and MXene to develop skin patch materials. These new composite materials offer a robust and functional structure for tissue engineering applications while also providing an innovative solution for wound healing processes. Future research will focus on optimizing the potential of these materials for broader biomedical applications and enhancing their functionality in a variety of clinical settings.

## Materials and Methods

### Biosynthesis and purification of BC

First, the bacterium *G. xylinum* (ATCC53582) is incubated in a static environment in HS (Hestrin–Schramm) medium. This incubation process allows the bacterium to actively multiply and produce BC [26]. The BC pellicles obtained are subjected to purification. This process aims to remove any contaminants present in the pellicles and to obtain the BC in a pure form. In the first purification step, the BC pellicles are immersed in 0.1 M NaOH solution and kept in this solution for 2 d. This process allows contaminants and bacterial residues on the surface of the pellicles to pass into the solution. The BC pellicles are then boiled in a mixture of 0.1 M NaOH solution and deionized water for 30 min. This boiling process ensures that the pellicles are cleaned, and bacterial cell debris is completely removed. BC pellicles are soaked in deionized water for 3 d after boiling to remove any residual NaOH. This process ensures that the pellicles are completely cleaned and purified. Finally, the purified BC pellicles are converted into a BC mixture using a tissue mixer. This mixture is then freeze-dried for 24 h using a lyophilized. This process ensures that the BC is pulverized and can be stored long-term. This detailed process starts from the initial BC production through the purification of the pellicles, and the final product, BC powder, is obtained.

### MXene (Ti_3_C_2_T*_x_*) synthesis

First, a sample containing 1 g of Ti_3_AlC_2_ was immersed in a 10-ml aqueous solution containing 49% hydrofluoric acid (HF). This step was carried out to allow the sample to interact with HF and to initiate the transition from Ti_3_AlC_2_ to Ti_3_C_2_T*_x_*. The immersion process was carefully continued at 60 °C for 30 h, during which time the sample was allowed to fully react with HF and the desired MXene product was formed. As a result of the immersion process, multilayered Ti_3_C_2_T*_x_*-MXene was obtained. This material was collected by centrifugation. Centrifugation was used to remove unwanted additives and excess HF solution. The speed of the centrifuge was set at 3,500 rpm, and the process time was 10 min. The obtained material was washed three times with ethanol and deionized water to remove the HF residue. This washing process was performed to remove HF residues and other additives in the product. At the end of the washing process, Ti_3_C_2_T*_x_*-MXene of the desired purity was obtained. Finally, the obtained Ti_3_C_2_T*_x_* powder was lyophilized at −50 °C for 24 h to obtain the final product. This process froze the product, allowing evaporation of water and permanent drying of the MXene powder. The final product was obtained as a layered MXene product in the desired form and purity. This process enabled the successful preparation of Ti_3_C_2_T*_x_*-MXene [27].

### Synthesis of BC film and BC/MXene (Ti_3_C_2_T*_x_*) composite hydrogel films

In the first step, 3 wt % BC solution is prepared by completely dissolving the dried BC mixture in a NaOH–urea–H_2_O system (w/w: 7:12:81) at low temperature. This process is carried out using intensive stirring, and as mentioned in previous studies, a transparent and viscous BC solution is obtained. In the second step, different amounts of the prepared Ti_3_C_2_T*_x_*-MXene are added to the BC solution to obtain 0.1, 0.2, 0.5, 1, and 2 wt % BC/MXene mixtures containing MXene. Finally, BC and BC/MXene composite hydrogel films are synthesized by chemical and physical double crosslinking. In this process, a crosslinker called ECH (Sigma) is used. ECH is added to BC and BC/MXene solutions in a certain ratio (BC:ECH = 4:4.2). After removal of air bubbles by centrifugation, the BC and BC/MXene solutions containing ECH are poured into a 24-well plate and kept at 4 °C for 24 h for the complete chemical crosslinking reaction to take place. The resulting BC-based films, after removal from the molds, are immersed in a 75% aqueous ethanol solution at 4 °C for 30 min to remove excess ECH and induce physical crosslinking. Then, it is immersed in 5% H_2_SO_4_ solution for 2 h to largely neutralize NaOH, and finally, it is immersed in deionized water for 7 d to completely remove excess ECH molecules and all small organic and inorganic molecules. This detailed process successfully synthesizes BC film and BC/MXene composite hydrogel films.

The production process of pure BC and BC/MXene is described step by step. First, there is the preparation step of Ti_3_C_2_T*_x_*-MXene powders. In this step, MXene powders are used as starting material. Then, the preparation of BC/MXene solution is started. In this step, MXene powders and BC material are combined and mixed for 5 h to obtain a homogeneous solution. In the third step, BC/MXene composite film is prepared. This process is carried out using ECH (epichlorohydrin) crosslinker. First, the BC/MXene solution is crosslinked with ECH at 4 °C for 24 h. Then, it is treated in 75% aqueous ethanol solution for physical crosslinking, and finally, BC/MXene composite hydrogel film is obtained. In the fourth and final step, BC film is prepared. First, the BC membrane is prepared and treated with aqueous NaOH/urea solution at −12 °C. Then, the mixture is freeze-dried, and the resulting BC solution is treated at 4 °C for 24 h using ECH crosslinker. Finally, it is treated in 75% aqueous ethanol solution for physical crosslinking to obtain the BC film.

### Mechanical property tests

Mechanical property tests were carried out to determine the strength, elasticity, and flexibility properties of BC-based composite hydrogel films. These tests were carried out using a universal machine tester, Instron 5567 (USA), to measure the compression and tensile capabilities of the hydrogels [28]. For compression tests, cylindrical composite hydrogel films with a diameter of 14 mm and a height of 14 mm were prepared. For tensile tests, membrane composite hydrogel films 5 cm long, 3 cm wide, and 3 mm thick were used. These standard dimensions were determined to ensure comparability of the test results. Compression and tensile tests were carried out at a speed of 1 mm/min at a rate up to 80% of the maximum tensile amount of the composite hydrogel films. The maximum compressive strength of the composite hydrogel films was determined by averaging five samples. The compressive strength and Young’s modulus of each sample were determined using the slope of the initial linear segment in the range of 0 to 25% of the stress–strain curves. To determine the elasticity and healing properties of the samples, four consecutive compression cycles were performed. These cycles were carried out up to 70% strain at the same rate to evaluate the elasticity and flexibility of the composite hydrogel films. Each sample was tested three times to ensure the reliability of the test results. These detailed mechanical property tests are important to understand and optimize the performance of composite hydrogel films for their application areas.

### Contact angle and water uptake capacity test

With the use of a water contact angle analysis equipment (DSA100, Krüss Company, Germany), the hydrophilicity of the composite hydrogel films was assessed. Deionized water was added to 200 ml of lyophilized, purified, and constructed BC-based composite films, which were then left at room temperature. Water droplets with a volume of 3 μl were dropped on the surface of BC and BC/MXene (Ti_3_C_2_T*_x_*) composite hydrogel films separately and then imaged with a camera at different time intervals to observe the state of the water droplets. An average of five composite hydrogel films was applied in each group. Equation 1 was used to compute water uptake capacity [29].

$$\text{Water uptake}\;\left(\%\right)=\left[\left(\mathrm{Ws}-\mathrm{Wd}\right)/\mathrm{Wd}\right]\times 100$$ (1)

where Ws and Wd stand for the weight of the original freeze-dried films and the weight of the swelled films at a specific time, respectively. For both groups, the analysis was done in triplicate.

The water absorption ability of BC-based composite hydrogel films was tested by soaking in phosphate-buffered saline (PBS; pH 7.4) at 37 °C. This test was performed to evaluate the interaction of composite hydrogel films with environmental fluids and the absorption of water through hydrogen bonds. First, freeze-dried BC and BC/MXene composite hydrogel films were placed in PBS and kept at 37 °C for a certain time. Then, the swollen samples were removed from the PBS at the specified time intervals and the solution on the surface was removed using filter paper. The composite hydrogel films were then immediately weighed to determine the amount of water uptake.

### Electrical conductivity measurement

Electrical conductivity measurements play a critical role in understanding the performance of BC and BC/MXene composite hydrogel films in various fields where they can be applied. These measurements determine the electrical conductivity levels of the composite hydrogel films, potentially assessing their suitability for use in electrically powered devices or energy storage systems. It is also important to understand how such nanomaterials can affect the performance of composite hydrogel films by assessing the effect of MXene content on electrical conductivity. These measurements were performed to understand the electrical properties of BC and BC/MXene composite hydrogel films, as well as the effect of different MXene contents on the electrical conductivity of composite hydrogel films. The data obtained can be used in material design and development to guide the design of optimized composite hydrogel films for future applications. In this way, electrical conductivity measurements provide an important tool to evaluate the potential applications of BC and BC/MXene composite hydrogel films and to optimize their future use in areas such as energy storage, sensor technology, or electronic devices. The electrical conductivity of BC films and BC/MXene (Ti_3_C_2_T*_x_*) composite hydrogel films was performed with samples in batches with different MXene content. In each batch, two probe methods were used, averaging five samples. It is a method used to measure electrical conductivity. In this method, the electrical resistance between two electrodes placed at the two ends of the sample is measured. The electrical conductivity of BC and BC/MXene composite hydrogel films with different MXene contents was measured by the two-probe method and averaged over five samples for each group. Briefly, composite hydrogel film samples (*l* = 3 mm, *d* = 12 mm) were soaked in PBS (pH 7.4) for 24 h before testing, and then two parallel titanium electrodes were fixed to the two ends of the composite hydrogel films. Their conductivities were determined using the potential state (CHI 660, USA). The electrical conductivity (σ, S/cm) value was calculated according to the following equation [28,30,31].

### Fourier transform infrared spectroscopy analysis

The structural details of the BC film and BC/MXene (Ti_3_C_2_T*_x_*) composite hydrogel films were performed using Fourier transform infrared spectroscopy (FTIR) (VERTEX 70, Bruker, Germany). The material FTIR spectra were measured from 500 to 4,000 cm^−1^ with a 5 cm^−1^ resolution.

### Thermogravimetric analysis and differential thermal analysis

In order to evaluate thermal stability of the BC and BC/MXene composite films, thermogravimetric analysis (TGA) of the composite hydrogel films was performed by measuring the decomposition temperature using TGA (TGA8000, TA Instruments, PerkinElmer, USA) under argon atmosphere using 5 mg of composite hydrogel films with a heating rate of 10 °C/min from room temperature to 600 °C. Composite hydrogel films were characterized using differential scanning calorimetry (DSC) analysis (Shimadzu model DSC-50 equipment) and programmed for room temperature heating (±20 °C) up to 120 °C for a period of 10 °C/min (initial run). The mass of the samples analyzed ranged from 5 to 10 mg.

### Scanning electron microscopy and transmission electron microscopy

The morphology of BC and BC/MXene composite hydrogel films was observed using a scanning electron microscope (SEM) (Quanta 200, FEI) at an accelerating voltage of 20.0 kV after sputter-coating with gold. The created MXene (Ti_3_C_2_T*_x_*) shapes, sizes, and interactions were examined morphologically using transmission electron microscopy (TEM). For the analysis (Hitachi HF-2000 TEM), 10 mg of powder was diluted in 20 ml of ethanol. The resulting suspension was then pipetted onto carbon-coated copper grit and allowed to dry for 5 min. Using a 200-kV voltage, the prepared grit was placed in the chamber on the imaging instrument’s column. The surface roughness and morphology of BC film and BC/MXene (Ti_3_C_2_T*_x_*) composite hydrogel films were evaluated by atomic force microscopy (AFM) using a NanoScope IV (Veeco, USA) model scanning probe microscope.

### X-ray diffraction analysis

For x-ray diffraction (XRD) (X’Pert3 Powder, PANalytical B.V., Netherlands) measurement with a Cu (copper; 1.54) inner wavelengths anode was used. The patterns were analyzed between 10 and 70° angle using 40-mA and 40-kV generator.

### X-ray photoelectron spectroscopy analysis

This analysis is performed using a technique called x-ray photoelectron spectroscopy (XPS) to evaluate the chemical states and binding properties of the elements present on the surface of the samples. The analysis was performed using a KRATOS AXIS-ULTRA DLD-600W XPS instrument located in Japan. This high-precision instrument sends x-rays by focusing on the sample surface and determines the elemental composition of the sample by analyzing the electrons emitted from the surface. Wide scans of the samples are performed in the 200- to 1,200-eV range. These scans ensure that all elements present on the surface of the samples are detected. Narrow scans of specific elements such as C 1s, O 1s, and Ti 2p have also been performed. These narrow scans were used to study the binding states and chemical states of specific elements in more detail. The data obtained provide in-depth information on the surface chemical states of the samples. For example, Ti 2p high-resolution spectra provide important information in determining the oxidation state and metallic character of Ti_3_C_2_T*_x_*-MXene. Similarly, C 1s spectra can show different binding states and oxidation levels of carbon. This analysis helps to gain a detailed understanding of the compositions and surface properties of Ti_3_C_2_T*_x_*-MXene and BC-based composite hydrogel films. It is also useful for identifying potential applications of the materials.

### In vitro biocompatibility

Cell studies were conducted at the Fatih Sultan Mehmet Foundation University’s BİORGİNE Biomaterials Nanotechnology Laboratory in compliance with ISO 10993-5 standards [32]. The biocompatibility properties of BC and BC/MXene composite hydrogel films were evaluated in the laboratory using enzymatic hydrolysis. This test was performed to determine the solubility and degradation rate of composite hydrogel films in the natural environment. First, a solution containing 0.5 g of cellulase was prepared. Cellulase is an enzyme used for enzymatic hydrolysis. It was obtained from Sigma-Aldrich. Enzyme activity is 370 U/g [33].

The cellulase solution was prepared by adding to 100 ml of HAc/NaAc buffer solution. It is a buffer solution used for the preparation of the enzyme solution. This solution is used to maintain a suitable pH level during the hydrolysis process. pH value is 4.8. During this process, the enzymatic degradation state of the composite hydrogel films was observed at regular intervals. Then, at certain time intervals, samples were taken from the enzymatic hydrolysis solution. These samples were then deproteinized using savage reagent. It is a reagent used for evaluation. This reagent is used to remove proteins from the hydrolysis solution and to measure the sugar content. It consists of a mixture of chloroform and n-butanol. The deproteinized enzymatic hydrolysis solution was used for the determination of total sugar and reduced sugar content. This detailed testing process was carried out to determine the biocompatibility profiles of the composite hydrogel films and to assess their suitability for biomedical applications.

### Determination of total sugar amount

The anthroin–sulfuric acid method for the determination of total sugar content is an effective spectrophotometric method for precisely measuring the amount of sugar in composite hydrogel films. This widely used method can detect not only glucose but also the presence of different types of sugars. In the first step, an anthroin sulfate solution is prepared; for this, 0.1 g of anthroin is added to 50 ml of concentrated sulfuric acid to form a solution. Then, 200 μl of the deproteinized enzymolysis solution was taken, 800 μl of anthroine sulfate was added to this solution, and the mixture was incubated in a boiling water bath for 10 min. This incubation time is sufficient to complete the reaction and ensure maximum color formation. The optical density of the mixture is then measured using a microplate reader at a wavelength of 620 nm. This measurement is used to indirectly determine the amount of sugar, while a standard curve is constructed for the determination of the total amount of sugar. For this purpose, a similar procedure is followed using 1 g/l glucose standard solution, and based on the standard solution, the total amount of sugar in the samples is calculated and the results are reported according to the standard curve. This detailed analysis provides important information on the biocompatibility and sugar content of composite hydrogel films. However, for a more comprehensive evaluation of the results obtained, it is important to assess the amount of sugars used in biocompatibility tests as well as the interactions of hydrogels with biological systems [34].

### Determination of the amount of reduced sugar in enzymolysis solution

Evaluation of the amount of reduced sugars obtained provides a deeper understanding of the bioavailability of hydrogels. This evaluation is important for understanding the interaction of hydrogels with biological systems and determining their potential in biomedical applications. In particular, the determination of the amount of reduced sugars can be an important indicator for understanding the solubility and degradation rate of hydrogels in biological environments. Furthermore, this information can help to assess the level of interaction of hydrogels with targeted biological tissues or systems and thus provide important clues in terms of biocompatibility and biological stability. However, evaluation of the amount of reduced sugars is also important for understanding the metabolic effects of hydrogels in biological systems. For example, the interaction of hydrogels with reduced sugars may have potential effects on glucose metabolism and energy production in biological systems. Determining these effects can help us better understand the long-term biological effects and biocompatibility of hydrogels. In conclusion, the determination of the amount of reduced sugars is seen as an important tool to evaluate the biological performance, bioavailability, and potential of hydrogels in biomedical applications. The amount of reduced sugars in the enzymolysis solution was determined using the 3,5-dinitrosalicylic acid (DNS) reagent method. This method is a widely used spectrophotometric technique for determining the presence and amount of reduced sugars and provides valuable information on the bioavailability of hydrogels. First, 400 μl of the deproteinized enzymolysis solution was taken and 600 μl of DNS reagent (Sigma-Aldrich) was added to it. This mixture was then incubated in a boiling water bath for 10 min to complete the reaction and color formation. The optical density of the mixture was then measured using a microplate reader (Thermo Fisher Scientific) at a wavelength of 540 nm. This measurement was performed to indirectly determine the amount of reduced sugar in the mixture. A standard curve was constructed to determine the amount of reduced sugar. For this purpose, a reduced sugar standard solution obtained by the same method was used. Based on this standard solution, the amount of reduced sugars in the samples was calculated and the results were reported according to the standard curve. This detailed analysis provides important information about the biodegradability of hydrogels and the amount of reduced sugars they contain [35].

### Cell culture and cytotoxicity evaluation

NIH3T3 (mouse embryonic fibroblast cell line) used in this study was obtained from the conservation center at Wuhan University for typical culture. The mouse embryonic fibroblast cell line NIH3T3 was obtained from the Type Culture Conservation Centre of Wuhan University. This cell line is a widely used model system in cell culture experiments. Cells were cultured in Dulbecco’s modified Eagle’s medium (DMEM) in an incubator at 37 °C in a humidified environment at 5% CO_2_ and 37 °C. It is the medium used in cell culture. This medium provides the necessary nutrients and conditions for proper growth and proliferation of cells. The culture medium was changed every 3 d. The components used in the culture medium are DMEM, 10% fetal bovine serum, and 1% penicillin/streptomycin. This medium was selected to provide optimum growth and proliferation conditions for the cells. In order for the cells to proliferate in a healthy manner and exhibit the desired phenotypes, it is important that they are regularly fed and kept in a suitable environment.

In the study, lactate dehydrogenase (LDH) activity in the culture medium released by NIH3T3 cells was determined to investigate the cytotoxicity of BC-based composite hydrogel films with different MXene contents. This evaluation was performed to assess the biotolerance of the composite hydrogel films and their possible harmful effects on cells. Prior to the tests, round samples of BC and BC/MXene composite hydrogel films, 7 mm in diameter and 2 mm thick, were treated with 75% ethanol for 2 h for sterilization and then washed with PBS (pH 7.4). Then, the purified hydrogels were immersed in a 24-well plate to reach an equilibrium swelling state in DMEM for 1 day at 37 °C. The 24-well plate is a type of plate used in cell culture. This plate combines many culture dishes into a single structure and provides a suitable environment for the growth and proliferation of cells. Subsequently, NIH3T3 cells were seeded on round samples of different composite hydrogel film samples at a density of 1 × 10^4^ cells per well each. On days 1 and 3 of incubation, the culture medium was collected, followed by centrifugation, and supernatant was taken. LDH activity was determined in the supernatant samples using Beyotime (China) LDH kit according to the manufacturer’s instructions. This kit contains the necessary reagents and instructions to perform spectrophotometric analysis of LDH. In this way, the effect of composite hydrogel films on cells and their cytotoxicity levels were evaluated. This detailed evaluation is important to assess the safety and efficacy of composite hydrogel films in biomedical applications. The results obtained have helped us to understand the effects of composite hydrogel film components on cells and to take the necessary precautions for their use in biomedical applications.

### Cell viability

Cell viability was determined using CCK-8 kit (Dojindo, Japan). It is a commercial kit used to assess cell viability. This kit is used to measure the metabolic activity of cells and determines cell viability through a reaction that changes the color of the cells. Briefly, in the contact assay, NIH3T3 cells (1 × 10^4^ per well) were cultured on round samples of sterilized BC-based films. The samples were 7 mm in diameter and 2 mm in thickness, and this culture was maintained for 1, 3, and 5 d in 96-well plates. Furthermore, extracts of all BC-based films for contact testing were obtained by immersion in DMEM for 2 d. These extracts were cultured with NIH3T3 cells (1 × 10^4^ per well) in 96-well plates for 1, 3, and 5 d. Each plate has 96 culture wells, and each well can contain a different experimental condition. Then, the culture medium was replaced with 100 μl of serum-free medium and 10 μl of CCK-8 solution, and this mixture was incubated for another hour at 37 °C in a CO_2_ incubator. The optical density of the medium was then measured in a microplate reader (Thermo Fisher Scientific) at a wavelength of 450 nm. Cell viability was calculated by Eq. 2.

$$(\%)\;\text{Cell viability}=[({\mathrm{OD}}_{S}-{\mathrm{OD}}_{B}/({\mathrm{OD}}_{N}-{\mathrm{OD}}_{B})\times 100$$ (2)

Here, OD_S_, OD_B_, and OD_N_ refer to the optical density values measured for the samples, blank control, and negative control, respectively. All samples were analyzed in triplicate to ensure the validity of the experimental data. Cell viability assays are a critical tool for assessing the biocompatibility and biotolerance of a material when cells interact with a material. In these experiments, optical density values reflecting the metabolic activity of cells are measured. Measurements are made by comparing the samples with standard media, called blank control and negative control. Negative control is a standardized medium that reflects the normal growth conditions of the cells. Samples are used to evaluate the reactions that occur as a result of the interaction of cells with the material. These samples are usually composed of various materials such as hydrogels and can be used for tests with or without contact with cells. To ensure the validity of the experiment, all samples are tested in triplicate. This approach increases the accuracy and reliability of the data. The calculation of cell viability (%) is based on the determination of the optical density values of the samples. This calculation is done by proportionally determining the optical density of the samples relative to the negative control and blank control values. These methods and calculations are important to objectively assess the effects of materials on cells.

### Hemolysis rate test

Blood compatibility assessment of BC-based films for various MXene contents was performed by hemolysis rate test. The test was performed based on previous literature. Briefly, round samples of different composite hydrogel films, 10 mm in diameter and 2 mm in thickness, were first washed three times in saline (0.9% NaCl solution). They were then immersed in brine in a 24-position plate for 24 h at 37 °C prior to testing. The samples were then transferred to 1.5-ml tubes containing 0.9 ml of saline in each 1.5-ml tube, and 0.1 ml of diluted whole blood of sheep (Vkan:Vsalt water = 1:1.25) was added to each tube. For positive and negative control, only distilled water and saline containing diluted whole blood were used, respectively. After incubation in a water bath at 37 °C for 1 h, each tube was centrifuged at 1,000 rpm for 5 min. The optical density of the supernatant was measured using a microplate reader (Thermo Fisher Scientific) at a wavelength of 545 nm. Hemolysis rate (HR) was calculated by Eq. 3:

$$(\%)\;\text{HR}=[({\mathrm{OD}}_{S}-{\mathrm{OD}}_{B}/({\mathrm{OD}}_{P}-{\mathrm{OD}}_{N})\times 100$$ (3)

Here, OD_S_, OD_B_, and OD_N_ refer to the mean absorbance values of the samples, negative control, and positive control, respectively. An average of six samples was taken for each group. The completion solution used for the hemolysis rate test is a standard solution containing 0.9% NaCl, and sheep whole blood is the target material of the test. Sheep whole blood is pre-prepared and diluted to a specific concentration for use in laboratory testing. The 1.5-ml tubes are used for storage and handling of test specimens and solutions. During testing, the optical density values of the different samples and controls are measured, and these values are used to determine the hemolysis levels of the samples. The negative control represents a nonhemolyzed environment, while the positive control represents an environment in which the whole blood is completely hemolyzed. Comparison of hemolysis levels between samples assesses the blood compatibility of the BC-based films tested. All samples are tested in triplicate, which ensures the validity of the experiment.

### Cell adhesion and spreading behavior studies

SEM and laser scanning confocal microscopy (LSCM; Olympus FV1000, Japan) were used to evaluate the adhesion and spreading behavior of NIH3T3 cells on different BC-based composite films. In this study, a combination of several microscopic techniques was used to visualize the adhesion and spreading patterns of cells on the film surface. First, NIH3T3 cells were added to sterilized BC-based film samples at a density of 5 × 10^4^ cells per well. It was observed how these cells attached to the film surface and how they spread. After culturing for 3 d, cells were washed with PBS (pH 7.4), then fixed with 2.5% glutaraldehyde, and dehydrated using a graded ethanol series. Subsequently, the samples were freeze-dried and prepared for SEM observation by liquefaction with gold. SEM was used to visualize the attachment patterns of the cells to the film surface and their spreading morphology with high-resolution images. Furthermore, LSCM was used to observe the interactions of the cells with the extracellular matrix inside. In this method, the structural organization of the F-actin cytoskeleton of the cells was examined by staining with tetramethyl rhodamine isothiocyanate (TRITC)-phalloidin (Sigma).

### Live/Dead Staining test

The proliferation, viability, early adhesion, and spreading ability of NIH3T3 cells on BC-based films were evaluated using the Live/Dead Staining kit. This assay was performed to determine the adhesion and growth behavior of the cells to the hydrogel surface. First, NIH3T3 cells were seeded into round samples (each 14 mm in diameter and 2 mm thick) of different sterilized BC-based films. Cells were seeded in a 24-well plate, each at a density of 5 × 10^4^ cells per well. After culturing for 1 day, cells were washed three times with PBS solution. Then, Live/Dead Staining kit was used to determine the viability and number of dead cells. For this purpose, calcein-AM (2 μM) and propidium iodide (PI) (4 μM) working solutions were added to the cells. Fluorescent dyes were used to determine live and dead cells. Calcein-AM stains live cells green, while PI stains dead cells red. These working solutions were prepared from stock solutions and added to the cells at 500 μl per well. Cells were incubated at 37 °C for 30 min. After incubation, the samples were washed with serum-free medium and analyzed under LSCM. In this way, the viability and spreading behavior of the cells were visually assessed. Furthermore, to determine the effect of electrostimulation, NIH3T3 cells were electrostimulated in pure BC film, BC/MXene (1%), and BC/MXene (2%) composite hydrogel films under various potentials for 30 min per day. At 3 d of incubation after electrostimulation, the viability and spreading behavior of the cells were again evaluated by Live/Dead Staining assay. These experiments were performed to understand the ability of cells to adhere and grow on the film surface, the biological compatibility of hydrogels, and the proliferation of cells on the composite hydrogel films.

### Methylthiazolytetrazolium assay

The MTT (methylthiazolytetrazolium) assay is a frequently used method for assessing the viability and proliferation of cells. This assay involves the use of MTT, an indicator, to determine the metabolic activity of cells. NIH3T3 cells were examined with this assay to evaluate the proliferation viability in pure BC film, BC/MXene (1%), and BC/MXene (2%) composite hydrogel films. In this experiment, the culture medium is replaced with a serum-free medium and MTT solution for 3 d after ES. MTT solution is reduced by the mitochondrial reductase enzymes of the cells to formazan crystals. These formazan crystals are then solubilized by adding dimethyl sulfoxide (DMSO). DMSO allows formazan to be released from the cells and dissolved so that the optical density can be measured. The optical density of the resulting solution is measured at a wavelength of 490 nm using a microplate reader. Optical density is directly related to the viability and proliferation of cells, so a higher optical density indicates more viable cells and greater proliferation. This experiment is a reliable method to assess the proliferation of cells in different types of composite hydrogel films. All samples are tested three times to ensure the reliability of the experiment.

### Quantitative real-time polymerase chain reaction

Expression levels of vascular endothelial growth factor, tissue growth factor-β (TGF-β), and epidermal growth factor genes were determined to analyze the wound healing process at the molecular level. These genes were targeted to evaluate the wound healing process. The expression levels of these genes were determined using quantitative real-time polymerase chain reaction (qRT-PCR). For this purpose, total RNA was extracted from samples in each treatment group (*n* = 4) using TRIzol reagent (Invitrogen, USA). Then, 1 μg of RNA was converted into complementary DNA (cDNA) using a reverse transcription reagents kit (Roche, Germany). The resulting cDNAs were analyzed by qRT-PCR using an Applied Biosystems 7500 Fast Real-Time PCR System (Applied Biosystems, USA). PCR was performed in 40 cycles with a specific temperature profile (2 min at 95 °C) after an initial activation step. Each cycle included denaturing the target gene at a specific temperature (3 s at 94 °C) followed by a primer extension step at a specific temperature (30 s at 60 °C), where the gene could be bound with specific primers. All PCRs were primers targeting predetermined gene regions. Primers were used to determine the expression levels of the target genes. Primer sequences and specifications are presented in Table 1. The data obtained were evaluated using the standard cycle threshold (Ct) method for analyzing the expression levels of the target genes. These analyses were performed to determine the expression differences between treatment groups of genes that contribute to the wound healing process.

**Table 1.** Primer sequences used for qRT-PCR

Primers	Direction	Ranking
VEGF	Forward	5′-TCACTATGCAGATCATGCGGA-3′
	Reverse	5′-GTCACTATGCAGATCATGCGGA-3′
TGF-β	Forward	5′-ATGACATGAACCGACCCTTC-3′
	Reverse	5′-TGTGTTGGTTGTAGAGGGCA-3′
EGF	Forward	5′-TCTGAACCCGGACGGATTTG-3′
	Reverse	5′-GACATCGCTCGCCAACGTAG-3′

## Results and Discussion

This study investigates the effect of different ratios of glucose (Glc) and d-saccharic acid potassium salt (KCl) on the growth of *G. xyliumm*, cellulose production, and film formation by thermal reduction of MXene. Within the scope of the study, various experiments were carried out using five different Glc ratios: 4:0, 3:1, 2:2, 1:3, and 0:4. Film formation was successfully achieved for all Glc ratios. This indicates that all combinations are suitable for cellulose production and film formation by *G. xyliumm*. The water content was 99.04 ± 0.21% at 4:0 ratio, 98.98 ± 0.31% at 3:1 ratio, 99.06 ± 0.37% at 2:2 ratio, 99.03 ± 0.15% at 1:3 ratio, and 99.40 ± 0.10% at 0:4 ratio (Table 2). The water content ratios show that the films have a very high water content, and this situation does not change much according to the ratios.

**Table 2.** Effect of different ratios of glucose (Glc) and d-saccharic acid potassium salt (SA) on the growth, cellulose production, and MXene formation by thermal reduction of film of *G. xyliumm*

	BC	BC	BC/MXene	BC/MXene	BC/MXene
Glc:SA	4:0	3:1	2:2	1:3	0:4
Water content (%)	99.04 ± 0.21	98.98 ± 0.31	99.06 ± 0.37	99.03 ± 0.15	99.40 ± 0.10
Yield (g/l)	3.17 ± 0.24	2.45 ± 0.13	2.61 ± 0.29	2.13 ± 0.17	1.14 ± 0.17

In terms of yield, the highest yield was obtained at 4:0 ratio (3.17 ± 0.24 g/l). This ratio is the ratio with the highest Glc content, and the yield decreases with decreasing Glc content. The yield was 2.45 ± 0.13 g/l at 3:1 ratio, 2.61 ± 0.29 g/l at 2:2 ratio, 2.13 ± 0.17 g/l at 1:3 ratio, and 1.14 ± 0.17 g/l at 0:4 ratio. These results show that Glc plays a critical role for cellulose production, and the yield increases with the increase in its ratio. In the cleaning and processing section of the table, visuals of the cellulose films obtained for each Glc ratio are presented. These images provide important data for visual evaluation of the films. In the thermal reduction of MXene section, color changes of MXene films were observed depending on different Glc ratios. MXene obtained at 4:0 ratio was dark brown, light brown at 3:1 ratio, orange at 2:2 ratio, yellow at 1:3 ratio, and light yellow at 0:4 ratio. These color changes indicate that thermal reduction at different ratios has a significant effect on the optical properties of MXene. The results of the present study reveal in detail the effects of different ratios of glucose and d-saccharic acid potassium salt on the growth of *G. xyliumm*, cellulose production, and film formation by thermal reduction of MXene. The results obtained show that specific Glc ratios have significant effects on cellulose production and optical properties of MXene.

### Mechanical properties

**Fig. 1.**
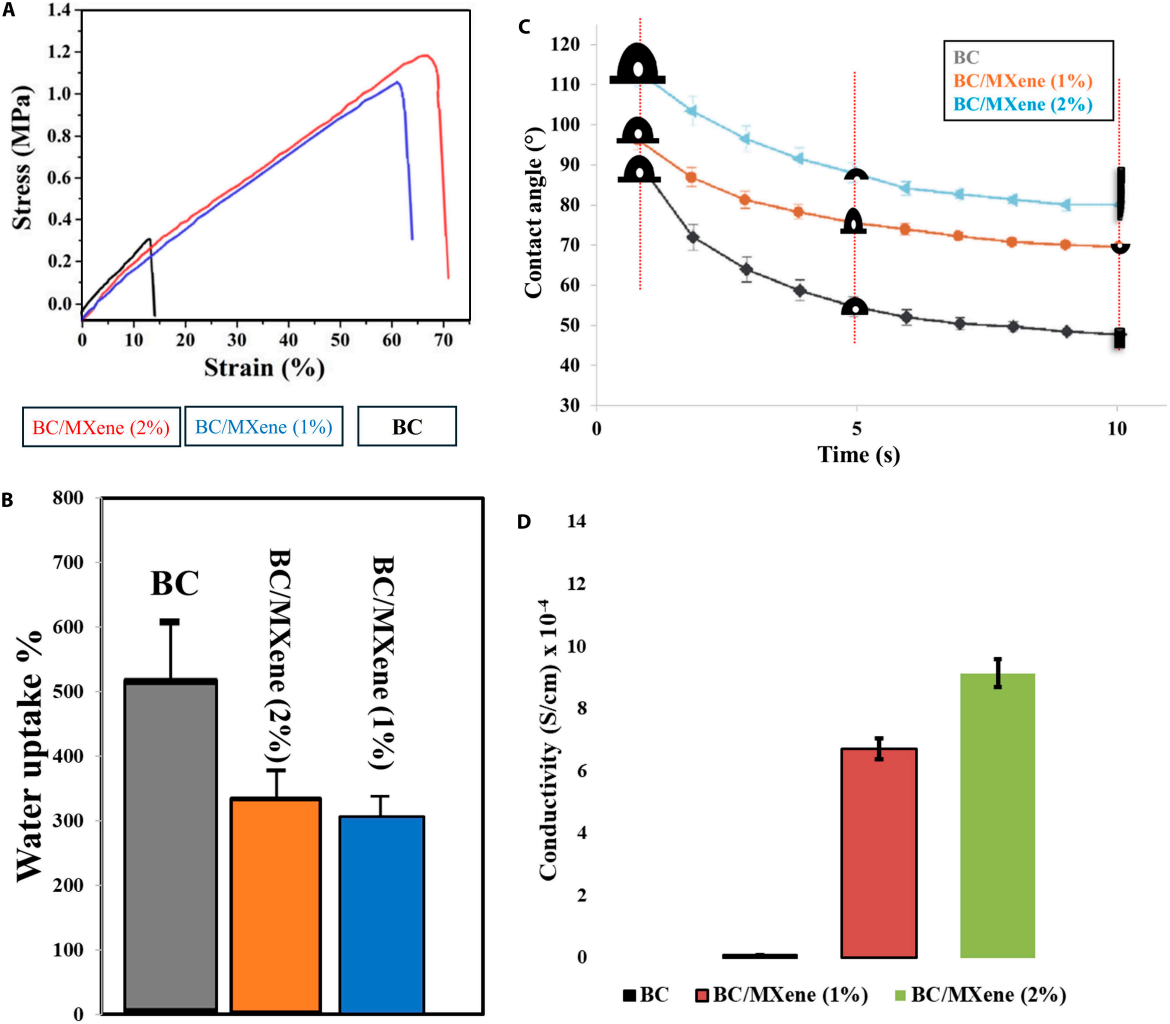
(A) Compression–strain curves of BC, BC/MXene (1 wt %), and BC/MXene (2 wt %) composite hydrogel films with varying maximum compression. (B) Water uptake capacity of BC and BC/MXene composite film samples. (C) Contact angle of BC and BC/MXene composite film soaked in PBS for 24 h. (D) Electric conductivity of BC, BC/MXene (1 wt %), and BC/MXene (2 wt %) composite films. Data are shown as mean ± SD (*n* = 5), and one-way analysis of variance (ANOVA) and Tukey's multiple comparison test were applied for analysis of significant difference.

Figure 1A presents the stress–strain curves of BC/MXene composite hydrogel films with different MXene contents. The graph compares the mechanical performances of BC film and BC/MXene composite hydrogel films containing 1 and 2 wt % MXene under maximum compression. The *x* axis shows the deformation percentage (%), and the *y* axis shows the stress (MPa) values. Pure BC film: This curve belongs to pure BC film. As the deformation percentage increases, the BC film deforms under a certain stress. The maximum stress value is around 0.3 MPa in the 50% to 60% deformation range. BC films show relatively lower mechanical strength since they do not contain MXene. BC/MXene (1%) composite film: This is the curve of BC/MXene composite hydrogel films containing 1 wt % MXene. As the deformation percentage of this composite hydrogel films increases, the stress value shows a higher increase compared to pure BC film. The maximum stress value is around 0.8 MPa at approximately 50% deformation. This shows that the presence of MXene in the BC matrix increases the mechanical strength of the composite hydrogel films. BC/MXene (2%) composite hydrogel films: This is the curve of the BC/MXene composite hydrogel films containing 2 wt % MXene. As the deformation percentage increases, this composite hydrogel film reaches the highest stress value. The maximum stress value is around 1.2 MPa at approximately 55 to 60% deformation. This shows that the mechanical strength of composite hydrogel films increases significantly with the increase of the MXene content. The graph clearly shows how the MXene content affects the mechanical properties of BC film. While the BC film shows the lowest mechanical strength, the mechanical strengths of the composite hydrogel films containing 1 and 2 wt % MXene increase. These results prove that the inclusion of MXene in the BC matrix increases the compressive strength of the composite hydrogel films, and this effect becomes more pronounced as the MXene ratio increases. These findings suggest that MXene may make an substantial contribution to the development of mechanically robust composite hydrogels [10,36]. It is known that wound dressings made from natural biopolymers such as alginate, collagen, and chitosan provide lower mechanical strength, while composite hydrogels offer higher durability compared to biopolymers [37,38]. However, the addition of nanomaterials such as MXene in the development of wound dressings substantially improves the mechanical properties of the hydrogel, allowing the creation of more durable and functional wound healing materials [39–41].

*σ*_Comp_, *ε*_Comp_, and *E*_Comp_ represent the stress at break, elongation at break, and modulus under compression, respectively. *σ*_Tens_, *ε*_Tens_, and *E*_Tens_ represent the tensile strength, elongation at break, and Young’s modulus under tension, respectively. Table 3 shows the room temperature compressive and tensile properties of BC and BC/MXene composite hydrogel films with various MXene contents. The table compares the stress at break, elongation at break, and modulus under compression/tension values ​​of these composite films. It clearly shows how the MXene content affects the mechanical properties of BC film. While the BC film shows the lowest mechanical strength, the mechanical strengths of composite hydrogel films containing 1 and 2 wt % MXene increase. These results prove that the incorporation of MXene into the BC matrix increases the compressive strength of the hydrogels, and this effect becomes more pronounced as the MXene content increases. These findings suggest that MXene may make a important contribution to the development of mechanically robust composite aerogels [42].

**Table 3.** Strength and tensile properties of BC, BC/MXene (1 wt %), and BC/MXene (2 wt %) composite hydrogel films with various MXene contents at room temperature (*n* = 4)

Samples	Compression			Tensile		
	*σ*_Comp_ (MPa)	*ε*_Comp_ (%)	*E*_Comp_ (MPa)	*σ*_Tens_ (MPa)	*ε*_Tens_ (%)	*E*_tens_ (MPa)
BC	0.98 ± 0.01	3.82 ± 0.25	71.98 ± 3.03	0.14 ± 0.02	66.24 ± 2.91	0.45 ± 0.12
BC/MXene (1 wt %)	1.44 ± 0.28	3.90 ± 039	70.93 ± 2.27	0.15 ± 0.02	62.83 ± 2.67	0.45 ± 0.10
BC/MXene (2 wt %)	1.20 ± 0.06	4.38 ± 026	70.57 ± 1.74	O.18 ± 0.03	56.31 ± 3.70	0.48 ± 011

### Contact angle and water uptake capacity

Figure 1B shows the water uptake percentage. This graph compares the water absorption capacities of BC and BC/MXene composite film samples. It is seen that BC film has the highest water absorption capacity (approximately 500%). The water absorption capacities of BC/MXene (1%) and BC/MXene (2%) composite film samples are around 300% and 320%, respectively. It is seen that the water absorption capacity of composite hydrogel films decreases as the MXene ratio increases.

The contact angle is shown in Fig. 1C. This graph shows the water contact angle of BC and BC/MXene composite film samples over time. A decrease in the contact angle is observed over time, i.e., the hydrogel surface becomes more wettable over time. The initial contact angle of the BC film is approximately 90°, which decreases to 60° over time. The initial contact angle of the BC/MXene (1%) composite film is approximately 100°, which decreases to 70° over time. The initial contact angle of the BC/MXene (2%) composite film is approximately 110° and decreases to 80° over time. It is observed that the initial contact angle of the hydrogel increases as the MXene ratio increases, but it becomes less wettable over time. Water absorption percentage, the high-water absorption capacity of the BC film, indicates that the composite hydrogel film structure has the ability to retain more water. This capacity decreases with the addition of MXene, which indicates that the MXene forms a more compact structure in the hydrogel structure and that water is less retained in this structure. The contact angle, on the other hand, is the lower initial contact angle of the pure BC film, indicating that its surface is more hydrophobic (non-water-attracting). The initial contact angle increases with the addition of MXene, indicating that the surface becomes more hydrophobic. Over time, the contact angles of all samples decrease, indicating that the surface becomes more hydrophobic as it comes into contact with water. These data indicate that the MXene ratio can be adjusted to optimize the interaction and mechanical properties of the BC/MXene composite hydrogel films with water [43,44].

### Electrical conductivity

**Fig. 2.**
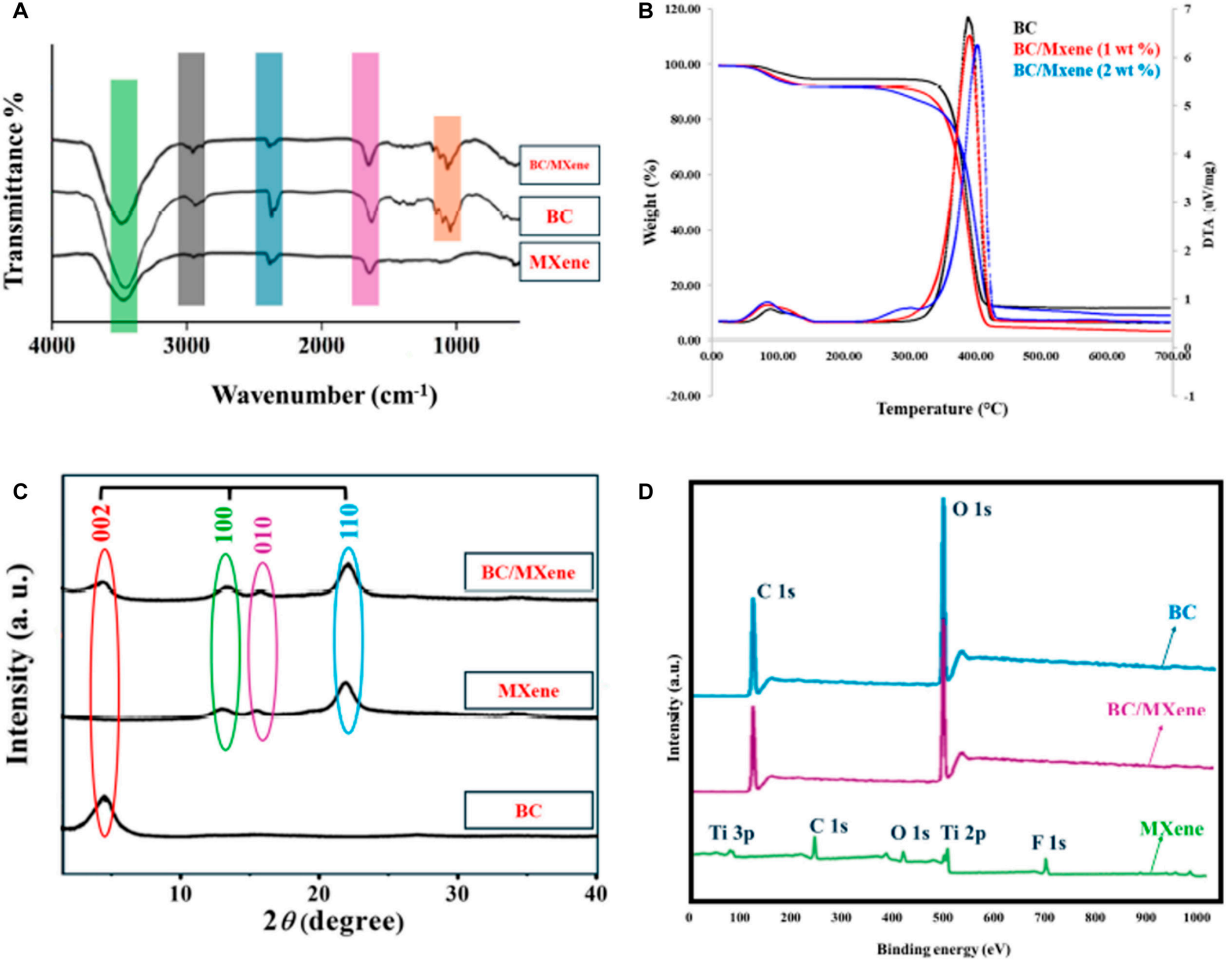
(A) FTIR spectra of Ti_3_C_2_T*_x_*-MXene, BC film, and BC/MXene composite film. (B) TG-DTA analysis of Ti_3_C_2_T*_x_*-MXene, BC film, and BC/MXene composite film. (C) XRD patterns of Ti_3_C_2_T*_x_*-MXene, BC film, and BC/MXene composite film. (D) XPS scanning spectra of Ti_3_C_2_T*_x_*-MXene, BC film, and BC/MXene (2%) composite film.

The electrical conductivity of BC, BC/MXene (1 wt %), and BC/MXene (2 wt %) composite hydrogel films are shown in Fig. 2D. The results show that BC has no electrical conductivity, while the electrical conductivity of BC/MXene composite hydrogel films increased depending on the MXene ratio. Among these composite films, BC/MXene (2%) film was observed to have the highest electrical conductivity and the best biocompatibility. It was observed that the electrical conductivity in BC films was 9.14 × 10^−4^ (S/cm) as the MXene concentration increased. Two-dimensional (2D) nanomaterials improve the conductivity and mechanical properties of composite hydrogel films because they have functional groups. The obtained BC/MXene (2%) composite film structure is suitable for use as “skin patch” in biomedical applications in terms of morphological, thermal, and water holding capacity and biodegradability.

### FTIR analysis

Figure 2A shows the FTIR spectra of BC film and BC/MXene composite hydrogel films with different MXene contents. FTIR spectra are an analysis method used to determine the chemical composition and functional groups of materials. There are three different spectra in the image: MXene FTIR spectrum: This spectrum is located at the bottom and represents the FTIR spectrum of only MXene powders. Various peaks are observed extending between 500 and 4,000 cm^−1^ on the wavelength axis. These peaks represent the characteristic functional groups and chemical bonds of MXene. BC FTIR spectrum: This spectrum in the middle shows the FTIR spectrum of the pure BC film. In this spectrum, characteristic peaks belonging to the structural features and functional groups of BC are observed. In particular, a broad OH stretching band around 3,000 cm^−1^ and various C–O and C–H bending bands in the range of 1,000 to 1,500 cm^−1^ are remarkable. BC/MXene composite film FTIR spectrum: This spectrum at the top represents the FTIR spectrum of the composite film formed by the combination of BC and MXene. In this spectrum, characteristic peaks belonging to both BC and MXene can be observed. This combination reveals the chemical presence and interactions of both components in the composite material. The 3,000 to 4,000 cm^−1^ range: Generally represents OH and NH stretching vibrations. In the BC and BC/MXene spectra, broad bands are observed in this region, indicating the presence of hydroxyl groups. The 2,000 to 3,000 cm^−1^ range: Contains C–H stretching vibrations [9], but no distinct peak is observed in these spectra. The 1,500 to 2,000 cm^−1^ range: May represent C=O and C=C stretching vibrations [45]. In the BC and BC/MXene spectra, distinct peaks can be found in this region. The 1,000 to 1,500 cm^−1^ range: Contains C–O and C–H bending vibrations [21]. In the BC and BC/MXene spectra, distinct peaks are found in this region. These FTIR spectra provide information about the chemical structures of BC and MXene and show that the BC/MXene composite film carries the chemical properties of both components. This proves that both components were successfully combined and coexisted during the synthesis of the composite material.

### TG-DTA analysis

Figure 2B shows the TGA and differential thermal analysis (DTA) results of BC-based composite hydrogel films with different MXene content. These analyses are used to evaluate the thermal stability and thermal behavior of the materials. Initial weight loss: In the first 100 °C, some weight loss is observed in all samples due to water loss. This is due to the evaporation of water or moisture in the materials. Major weight loss: In the range of approximately 300 to 400 °C, a large weight loss is observed in all samples. This represents the thermal degradation of the main structure of the materials. In BC/MXene samples (especially those with 2 wt % MXene content), weight loss starts at a higher temperature compared to pure BC. This indicates that the addition of MXene increases the thermal stability of BC. Final weight: After approximately 600 °C, the weight change becomes stable in all samples and the amount of residue is observed at this point. The amount of residue increases as the MXene content increases.

Endothermic–exothermic reactions in DTA analysis; a large endothermic peak is observed in the range of 300 to 400 °C. This indicates the thermal decomposition process of BC and BC /MXene samples. As the MXene content increases, the temperature of this reaction increases slightly, indicating that MXene increases the thermal stability of BC. Thermal behavior of BC: Pure BC experiences a serious weight loss at approximately 300 °C and undergoes thermal decomposition [46,47].

The effect of MXene in this study: The addition of MXene increases the thermal stability of BC. This is confirmed by the fact that the weight loss starts at higher temperatures and the temperature of the endothermic reaction increases in DTA analysis. The amount of residue: As the MXene content increases, the amount of residue remaining after thermal decomposition also increases. These analyses show that the addition of MXene significantly increases the thermal stability of BC-based composite films.

Table 4 shows the degradation temperature (Td) and loss weight (Delta W) values ​​of BC and BC/MXene composite hydrogel films with different MXene content according to TGA results. Studied composite hydrogel film samples (BC and BC film enriched with various MXene contents). Td (°C): Degradation temperature. It expresses the temperature at which the composite hydrogel films begin to thermally degrade. Delta W (%): Weight loss. It expresses the percentage of weight lost by composite hydrogel films as a result of thermal degradation.

**Table 4.** Degradation temperature (Td) and loss weight (Delta W) of BC and BC/MXene composite hydrogel films with different MXene content based on TGA analysis

Samples	Td (°C)	Delta W (%)
BC	343.33	88.79
BC/MXene-1%	351.56	67.67
BC/MXene-2%	352.76	54.66

The degradation temperature of pure BC was determined as 343.33 °C. The degradation temperature of the BC/MXene composite film with 1% MXene content increased to 351.56 °C. The degradation temperature of the BC/MXene composite film with 2% MXene content increased to 352.76 °C. As the MXene content increases, the degradation temperature of the composite hydrogel films increases. This means that MXene increases the thermal stability of BC film [48,49].

The weight loss percentage of pure BC due to thermal degradation was determined as 88.79%. This value decreases to 67.67% in the BC/MXene (1%) composite film. It decreases to 54.66% in the BC/MXene (2%) composite film. As the MXene content increases, the lost weight of the composite hydrogel films decreases, indicating that MXene increases the thermal stability of BC films. Thermal stability: The addition of MXene increases the thermal stability of BC films, meaning that the composite hydrogel films begin to decompose at higher temperatures.

Weight loss: As the MXene content increases, the weight lost by composite hydrogel films due to thermal degradation decreases. This indicates that MXene strengthens the BC structure and increases thermal durability. These results indicate that MXene improves the thermal properties of BC films and potentially more durable composite materials can be obtained. The thermal behaviors of pure BC and BC/MXene composite hydrogel films were investigated in detail using TGA. In the thermal analysis of the pure BC film, an initial significant weight loss was observed around 100 °C. This weight loss is due to the evaporation of the absorbed moisture and hydrogen-bonded water molecules in the film. At higher temperatures, a second weight loss phase was observed between 100 and 400 °C. The weight loss in this range reached a maximum at 343.33 °C, which was associated with the thermal decomposition, depolymerization, and disintegration of BC glucosyl units and free radicals. The formation of a charred residue also occurred during this process. In BC/MXene composite hydrogel films, thermal analysis results showed that the weight loss phases shifted to higher temperatures. In addition, it was observed that the amount of weight loss (delta weight) decreased as the MXene content increased. These findings show that MXene increases the thermal stability of BC/MXene composite hydrogel films. The mechanism by which MXene increases thermal stability can be explained by its ability to protect cellulose chains against thermal shock. MXene delays the degradation of cellulose at high temperatures, causing degradation to occur at higher temperatures and reducing weight loss. In this way, BC/MXene composite hydrogel films become resistant to higher temperatures and their thermal stability increases. The BC/MXene composite hydrogel films with high thermal stability (up to 300 °C) maintain structural stability after sterilization and demonstrate superiority in infection control. These properties play a critical role in smart applications, such as biosensors and temperature-controlled drug delivery systems, especially for long-term and safe wound care [13,39].

### SEM and TEM analysis

**Fig. 3.**
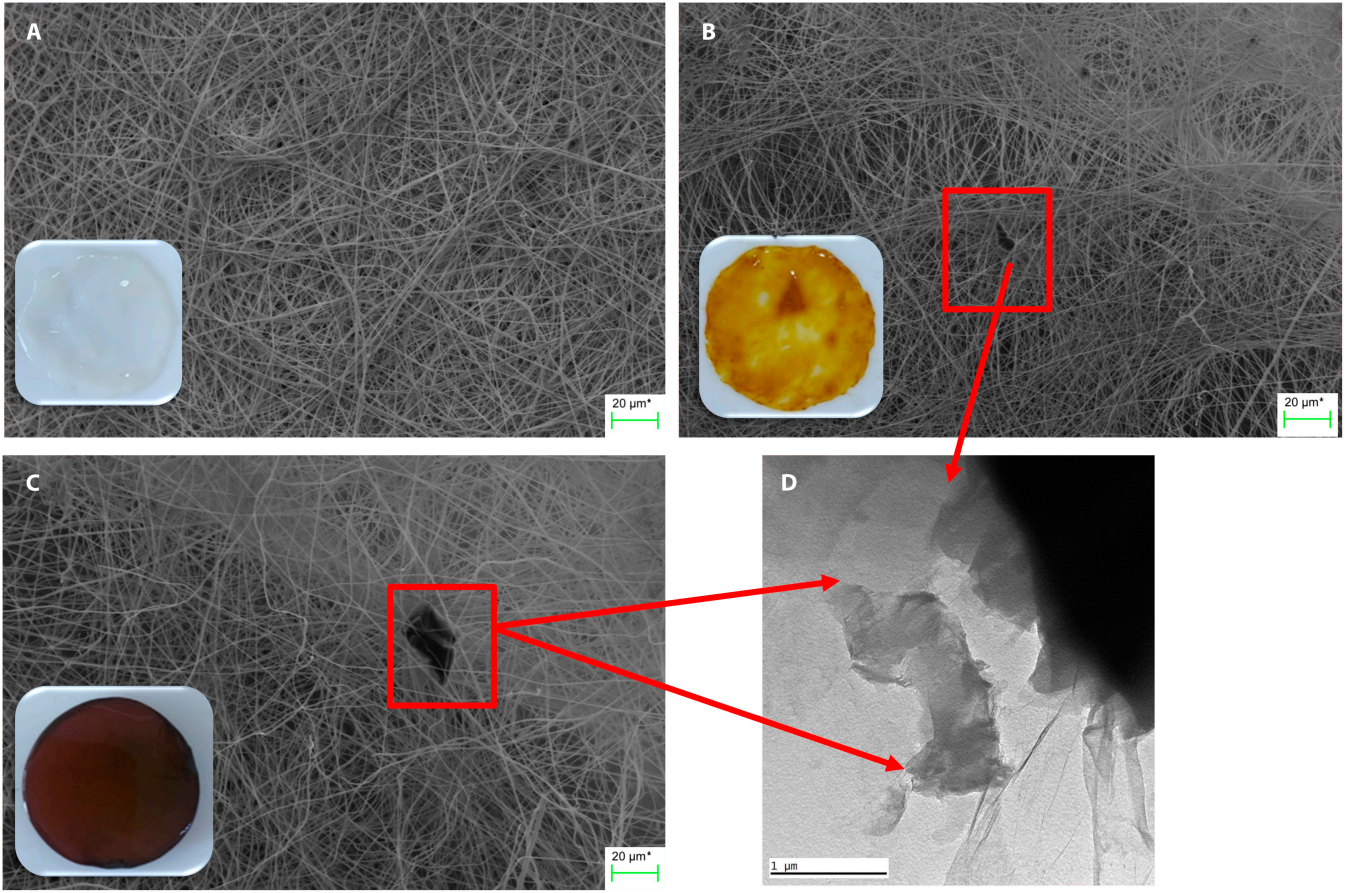
(A) SEM and optical microscopy image of BC film (transparent). (B) SEM morphological images of BC/MXene (1%) composite film and optical microscopy images (yellow). (C) SEM and optical microscopy images of BC/MXene (2%) composite film (dark brown). (D) TEM morphological images of MXene structures loaded on BC.

Figure 3 shows the image obtained by SEM analysis of the top surface of BC film (gray, Fig. 3A), BC/MXene (1 wt %) composite film (yellow, Fig. 3B), and BC/MXene (2 wt %) composite film (dark brown, Fig. 3C). As observed, BC has a random and dense nanofibril network structure, which has formed a highly porous structure allowing high liquid retention. The diameter of pure BC film fibrils was measured between 52 and 98 nm in previous study (ImageJ software) [7].

It includes two different images of Ti_3_C_2_T*_x_*-MXene. Figure 3B and C shows the macroscopic view of Ti_3_C_2_T*_x_*-MXene. It appears as a black or dark colored powdery substance inside the reduced BC fibril structure. Figure 3D clearly shows the typical layered structure of Ti_3_C_2_T*_x_*-MXene in the TEM image. Thin sheets or leaf-like structures stacked in layers are noticeable. Such structures are critical to understanding the properties of MXene materials. The scale bar in the SEM image helps us understand the dimensions of the details in the image. This scale bar is 20 μm long and indicates that the image is magnified approximately 100,000 times. This allows us to observe the fine details of the material’s nanostructure. Figure 3A presents the macroscopic view of the BC nanofibril material, while Fig. 3B shows the microscopic structure of both BC and the coating MXene (1 wt %) to BC, indicating the embedding of MXene in the matrix and its placement on the surface. The image shows SEM images of the surface, cross-section, and pore walls of BC/MXene composite hydrogel films with different MXene contents. Detailed examinations of the surface, cross-section, and pore walls were performed for each composite film sample. These SEM micrographs show the microstructural features of BC/MXene composite hydrogel films containing different concentrations of MXene and the distribution of MXene in the matrix. The images reveal that MXene can be homogeneously distributed in the BC matrix and can be located both on the surface and in the pore walls. Such detailed microstructural analyses are critical in understanding the performance and functionality of composite materials [50–52].

### XRD analysis

Figure 2C shows the XRD patterns of Ti_3_C_2_T*_x_*-MXene, BC, and BC/MXene (2%) composite films. XRD patterns provide information about the crystal structure of the material and contain peaks corresponding to crystal planes specific to a particular material. The intensity (*y* axis) and 2θ (degree) (*x* axis) are given in the graph. XRD patterns of three different samples are seen in the graph. When BC was formed with MXene and composite films, Miller index patterns were observed at 002, 100, 010, and 110 to the right, respectively. Peaks are related to the distances between certain planes of the crystal structure. These peaks are usually defined by “hkl” indices. Here, peaks corresponding to certain “hkl” planes are marked with different colors. The peak positions and intensities of each pattern are important for understanding the crystal structure and composition of the material. As can be seen from the graph, the BC/MXene mixture shows a combination of peaks specific to BC and MXene [53–55].

### XPS analysis

Figure 2D shows the XPS scanning spectra of Ti_3_C_2_T*_x_*-MXene, BC film, and BC/MXene-2% composite film. XPS is used to analyze the surface chemistry and elemental distribution of the materials. The horizontal axis in the graph shows the binding energy (eV), and the vertical axis shows the intensity. Peak signals of different elements can be observed for each sample. Ti_3_C_2_T*_x_*-MXene spectrum: In this spectrum, Ti (titanium) 2p, C (carbon) 1s, and O (oxygen) 1s peaks are clearly observed. The Ti 2p peak is located around 460 eV and represents titanium, the main component of MXene. The O 1s peak is observed around 530 eV, indicating oxygen groups on the MXene surface. The C 1s peak is located around 285 eV. BC/MXene-2% composite film spectrum: This spectrum shows the BC/MXene-2% composite film. In this spectrum, the characteristic peaks of both BC and MXene can be seen. Ti 2p, C 1s, and O 1s peaks are also found here. However, the intensity and peak shapes are different from pure MXene because the effect of BC is also present in this spectrum. Spectrum: In the BC film spectrum, C 1s and O 1s peaks are prominent. These peaks represent the main components of BC, carbon, and oxygen groups. The Ti 2p peak is not found in this spectrum because BC film does not contain titanium. When all three spectra are examined together, the characteristic Ti, C, and O peaks of Ti_3_C_2_T*_x_*-MXene are also present in the BC/MXene (2%) composite film. However, only carbon and oxygen peaks are found in the BC film. These differences indicate that the spectral properties of MXene change when combined with BC and both components affect the surface chemistry of the composite film. In summary, these XPS spectra provide important information to understand the surface chemistry of the BC/MXene-2% composite film. The characteristic peaks of Ti_3_C_2_T*_x_*-MXene and the effect of BC provide analysis of the elements and chemical bonds present on the surface of the composite hydrogel [55–57].

### In vitro biocompatibility and cell line

**Fig. 4.**
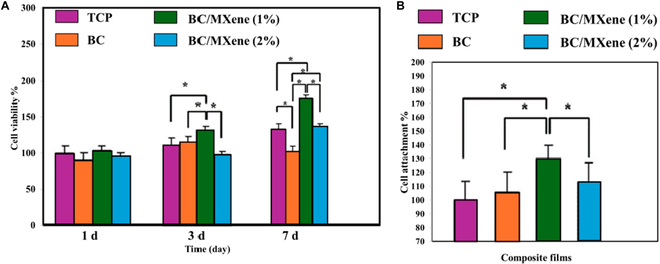
(A) Cell viability of TCP, BC, BC/MXene 1%, and BC/MXene 2% composite films. (B) Attachment percentages of NIH3T3 cells in TCP, BC, BC/MXene 1%, and BC/MXene 2% composite films. Data are shown as mean ± SD (*n* = 7), and one-way ANOVA and Tukey's multiple comparison test were applied for analysis of significant difference.

Cell viability rates are close to each other on the first day, and no important difference is observed. Differences begin to emerge between cell viability rates on the third day. BC/MXene (%1) sample has the highest viability rate. On the seventh day, BC/MXene (%1) sample again has the highest cell viability rate, significantly higher than BC/MXene (%2) and other samples. (*) marks indicate statistically significant differences. In Fig. 4B, BC/MXene (%1) sample has the highest cell adhesion percentage and significantly higher than all other samples.

BC and BC/MXene (%2) composite hydrogel film samples show similar values ​​in terms of cell adhesion percentage, but significantly lower compared to BC/MXene (%1) samples.

Figure 4A shows the effect of BC and BC/MXene mixtures on cell viability. While similar cell viability was observed in all samples on the first day, the BC/MXene (%1) composite hydrogel film sample had the highest cell viability on the third day. The seventh day data show that the BC/MXene (%1) sample supported cell viability the most. Adding MXene at low rates increases the biocompatibility and cell viability of the BC film. However, increasing the MXene rate to 2% negatively affects cell viability. Figure 4B shows how BC and MXene mixtures affect the ability of cells to adhere to the surface. The BC/MXene (%1) composite hydrogel film sample has the highest cell adhesion rate, indicating that the hydrogel provides a suitable surface for cell adhesion [21,58–60]. While tissue culture plastic (TCP) and BC samples have lower cell adhesion rates, the BC/MXene (%2) composite hydrogel film sample also shows a relatively lower cell adhesion percentage. This shows that adding MXene at low rates increases cell adhesion but adding it at high rates eliminates this advantage. These data suggest that the BC/MXene composite hydrogel films have potential use in biomedical applications, particularly in areas such as cell culture and tissue engineering. The addition of MXene in the correct proportions increases the biocompatibility and cell-supporting properties of the hydrogel [43,61]. Generally, a hemolysis ratio (HR) of <5% is considered permissible for biomaterials. The HR value of the positive control and the negative control was 100% and 0%, respectively, and the HR ratios of the BC films, BC/MXene (1%), and BC/MXene (2%) composite hydrogel films were 0.09%, 0.86%, and 1.49% from the pure BC films to the BC/MXene (2%) composite film, respectively, implying good blood compatibility and no hemolysis. Therefore, BC-based composite films, especially BC/MXene (2%) composite hydrogel films, possess good biocompatibility.

**Fig. 5.**
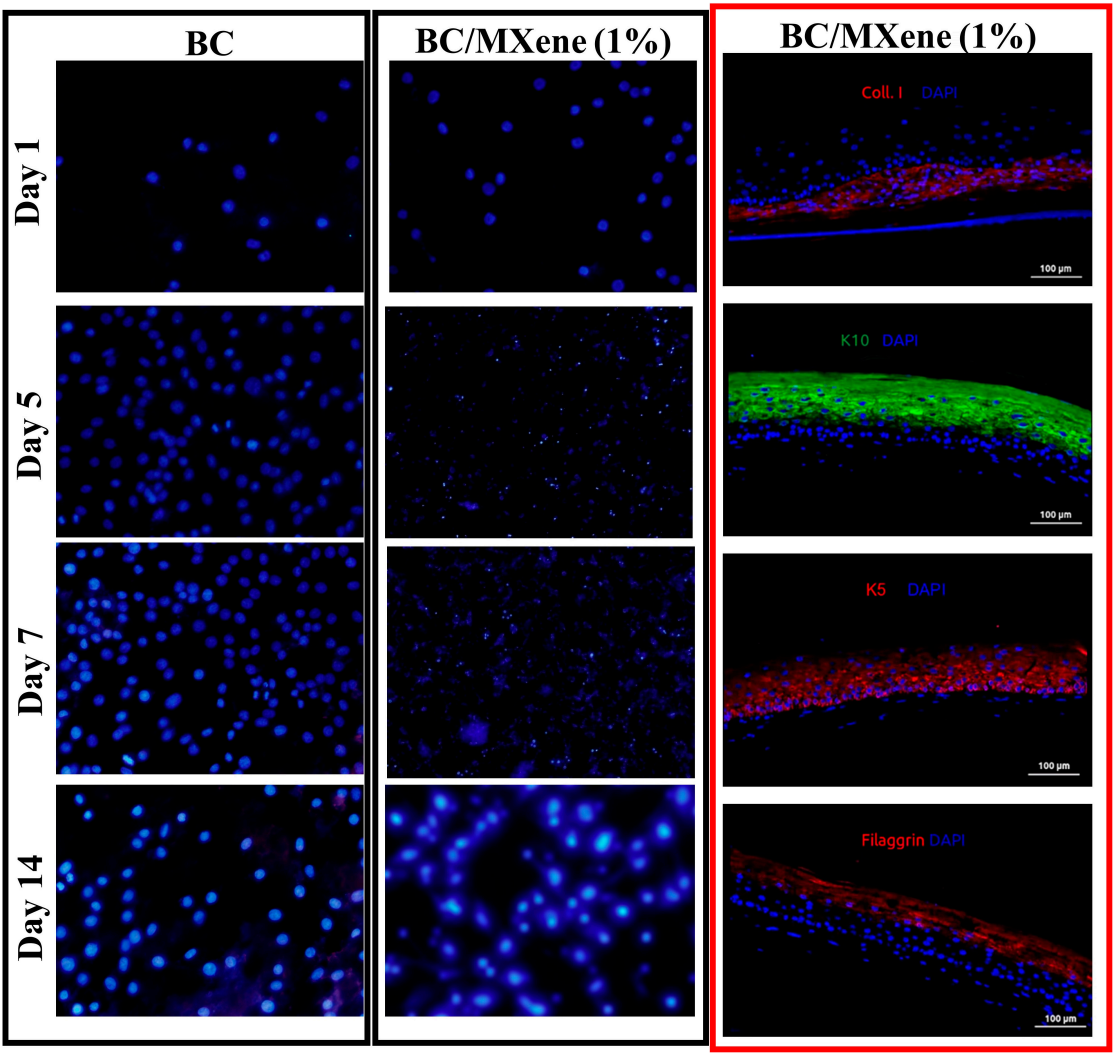
LSCM images of NIH3T3 cells after 24-h culture on BC and BC/MXene 1% composite hydrogel films. Scale bars are 100 μm for all images.

Figure 5 shows the growth of NIH3T3 cells on BC and BC/MXene (1%) samples on various days. Each image contains images taken at different times (days 1, 5, 7, and 14). The cell nuclei are marked in blue with 4′,6-diamidino-2-phenylindole (DAPI) stain in the images. Then, there are images of staining with specific protein markers in BC/MXene composites containing 1% MXene. A different protein (collagen I, K10, K5, filaggrin) is shown in each image, and the cell nuclei are marked in blue with DAPI stain. These proteins were used to study the characteristics of the cells such as differentiation and matrix formation. Collagen I: Collagen type I is generally associated with extracellular matrix and connective tissue. Stained in red. K10: Keratin 10, a marker of epidermal cell differentiation. Stained in green. K5: Keratin 5 is associated with the basal cell layer and epidermal stem cells. Stained in red. Filaggrin: Filaggrin is an important protein in the epidermal differentiation and keratinization process. Stained in red. Day 1 [BC versus BC/MXene (1%)]: Cell density was low in both groups. This indicates that the cells had just started the culture and were still in the process of adhesion and spreading to the surface. Day 5 [BC versus BC/MXene (1%)]: Cell density increased in the BC group, while it was lower in the BC/MXene (1%) group. The addition of MXene may have negatively affected cell proliferation. Day 7 [BC and BC/MXene (1%)]: Cells appeared more widespread and denser in the BC group, while they were sparser in the BC/MXene (1%) group. This suggests that MXene may have an inhibitory effect on cell growth. Day 14 [BC versus BC/MXene (1%)]: Cell density appeared to have reached its maximum level in the BC group, while the cell density was still low in the BC/MXene (1%) group. This may indicate that MXene addition has negative effects on cell growth in the long term. According to marker staining [BC/MXene (1%)] results: Collagen I: Collagen type I staining indicates the ability of cells to produce matrix. Collagen production is important for tissue engineering and wound healing. K10: Keratin 10 staining indicates the degree of differentiation of epidermal cells. K5: Keratin 5 staining indicates the presence of a basal cell layer and stem cell-like properties of cells. Filaggrin: Filaggrin staining indicates that epidermal cells are in the process of terminal differentiation [21,60]. These images comparatively show the growth and differentiation processes of NIH3T3 cells in BC and BC/MXene (1%) composites. MXene addition may have had a negative effect on cell proliferation, but more detailed analyses are needed on how it affects the production of specific proteins. Such studies are critical for biomaterial development and understanding cellular responses.

## Conclusion

The results of this study indicate that MXene addition to BC films has a notable effect on NIH3T3 cell growth and differentiation. Over a period of 14 d, the BC/MXene (1%) composite showed reduced cell proliferation compared to the BC group, suggesting that MXene may inhibit cell growth, particularly over longer durations. Despite this, protein marker staining revealed that cells in the BC/MXene (1%) composite film maintained the ability to produce essential proteins such as collagen I, K10, K5, and filaggrin, which are vital for extracellular matrix formation, cell differentiation, and tissue regeneration. This indicates that while MXene may affect cell proliferation, it does not entirely inhibit key cellular functions related to tissue engineering. However, the inhibitory effect on cell proliferation underscores the need for further investigation to understand how MXene affects both cellular behavior and specific protein expression. Optimizing MXene concentrations and refining composite properties could enhance the material’s potential in biomedical applications. It is expected to provide safe and effective performance, especially in tissue engineering applications that require long-term contact, such as skin patches.

## Data Availability

The study is completely new, and all data and photos are shared in this study.
